# The complete chloroplast genome of *Eclipta prostrata* L. (Asteraceae)

**DOI:** 10.1080/23802359.2016.1176882

**Published:** 2016-06-20

**Authors:** Jee Young Park, Yun Sun Lee, Jin-Kyung Kim, Hyun Oh Lee, Hyun-Seung Park, Sang-Choon Lee, Jung Hwa Kang, Taek Joo Lee, Sang Hyun Sung, Tae-Jin Yang

**Affiliations:** a Department of Plant Science, College of Agriculture and Life Sciences, Plant Genomics and Breeding Institute, and Research Institute of Agriculture and Life Sciences, Seoul National University, Seoul, Republic of Korea;; b Phyzen Genomics Institute, Seongnam-Si, Gyeonggi-Do, Republic of Korea;; c Hantaek Botanical Garden, Yongin, Gyeonggi-Do, Republic of Korea;; d College of Pharmacy and Research, Institute of Pharmaceutical Science, Seoul National University, Seoul, Republic of Korea;; e GreenBio Science and Technology, Crop Biotechnology Institute, Seoul National University, Pyeongchang, Republic of Korea

**Keywords:** Chloroplast, *Eclipta prostrata*, genome sequence

## Abstract

*Eclipta prostrata* is an herbal medicinal plant belonging to the Asteraceae family. In this study, complete chloroplast genome sequence of the *E. prostrata* was characterized by *de novo* assembly using whole genome sequence data. The genome of *E. prostrata* was 151,757 bp in length, which was composed of large single copy region of 83,285 bp, small single copy region of 18,346 bp and a pair of inverted repeat regions of 25,063 bp. The genome harboured 80 protein coding sequences, 30 tRNA genes and 4 rRNA genes. We confirmed close taxonomic relationship between *E. prostrata* and *Helianthus annuus* through phylogenetic analysis with chloroplast protein-coding genes.

*Eclipta prostrata* is an annual herbal plant species belonging to the Asteraceae family, which is mainly distributed in tropical and subtropical regions in the worlds (Panero et al. 1999). *E. prostrata* has been used as a folk medicine for treatment of inflammatory diseases such as hepatitis (Han et al. 1998). In addition, pharmacological studies reported various biological activities of *E. prostrata,* including anti-inflammatory and anticancer-cytotoxic activity (Arunachalam et al. 2009; Khanna & Kannabiran 2009; Liu et al. 2012). However, there is rare genetic and genomic study for authentication and breeding of these plants. In this study, we characterized chloroplast genome sequence of *E. prostrata* to provide useful genome information for this plant species.

To determine chloroplast genome sequence, we extracted total genomic DNA from leaves of *E. prostrata* which were provided by HanTaek Botaniccal Garden (Yongin, Korea, www.hantaek.co.kr). The extracted DNA was used to construct Ilumina paired-end (PE) genomic library and sequenced using an Illumina MiSeq platform (Illumina, San Diego, CA) in Lab Genomics Co. (Seongnam, Korea; http://www.labgenomics.co.kr) . High quality PE reads of ∼1.4 Gb were assembled by CLC genome assembler (ver. 4.06 beta, CLC Inc, Aarhus, Denmark) according to the previous study (Kim et al. 2015a, b), and then representative chloroplast contigs were retrieved, ordered and combined into a single draft sequence by comparing chloroplast genome sequence of *Centaurea diffusa* (KJ690264). The draft sequence was manually corrected by PE read mapping. The genes in the chloroplast genome were annotated using the DOGMA program (Wyman et al. 2004) and manual curation based on BLAST searches.

The complete chloroplast genome of *E. prostrata* (KU361242) was 151,757 bp in length, which was composed of large single copy region of 83,285 bp, small single copy region of 18,346 bp and a pair of inverted repeat regions of 25,063 bp, as typical chloroplast structure. A total of 114 genes were predicted in the genome, including 80 protein-coding genes, 30 tRNA genes and 4 rRNA genes.

Phylogenetic analysis was performed using common 68 protein-coding genes in 11 chloroplast genomes by maximum likelihood method in the MEGA 6.0 (Tamura et al. 2013) for *E. prostrata* and related 10 species belonging to the Asteraceae family. The phylogenetic tree showed that *E. prostrata* was grouped with other two species in Heliantheae tribe, in which *E. prostrata* was placed more closely to *Helianthus annuus* (sunflower) (Figure 1).

**Figure 1.**
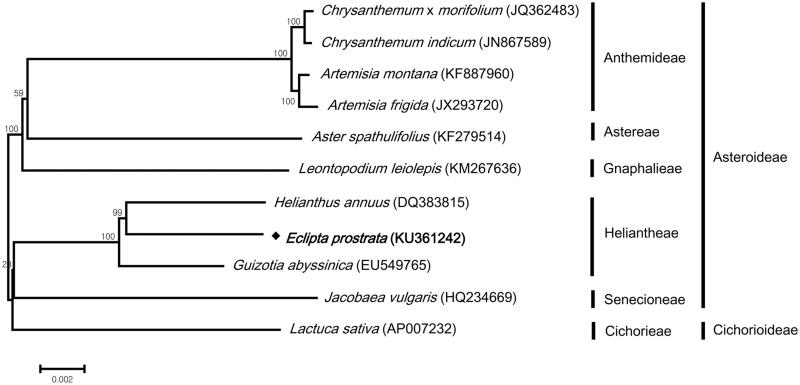
Phylogenetic tree showing relationship between *Eclipta prostrata* and 10 species belonging to the Asteraceae family. Phylogenetic tree was constructed based on 68 protein-coding genes of chloroplast genomes using maximum likelihood method with 1000 bootstrap replicates. Numbers in each the node indicated the bootstrap support values.
